# Cough Sound Detection and Diagnosis Using Artificial Intelligence Techniques: Challenges and Opportunities

**DOI:** 10.1109/ACCESS.2021.3097559

**Published:** 2021-07-15

**Authors:** Kawther S. Alqudaihi, Nida Aslam, Irfan Ullah Khan, Abdullah M. Almuhaideb, Shikah J. Alsunaidi, Nehad M. Abdel Rahman Ibrahim, Fahd A. Alhaidari, Fatema S. Shaikh, Yasmine M. Alsenbel, Dima M. Alalharith, Hajar M. Alharthi, Wejdan M. Alghamdi, Mohammed S. Alshahrani

**Affiliations:** Department of Computer ScienceCollege of Computer Science and Information TechnologyImam Abdulrahman Bin Faisal University 48023 Dammam 31441 Saudi Arabia; Department of Networks and CommunicationsCollege of Computer Science and Information TechnologyImam Abdulrahman Bin Faisal University 48023 Dammam 31441 Saudi Arabia; Department of Computer Information SystemsCollege of Computer Science and Information TechnologyImam Abdulrahman Bin Faisal University 48023 Dammam 31441 Saudi Arabia; Department of Emergency MedicineCollege of MedicineImam Abdulrahman Bin Faisal University 48023 Dammam 31441 Saudi Arabia

**Keywords:** Artificial intelligence (AI), cough detection, 2019 novel coronavirus disease (Covid-19), respiratory illness diagnosis, cough-based diagnosis

## Abstract

Coughing is a common symptom of several respiratory diseases. The sound and type of cough are useful features to consider when diagnosing a disease. Respiratory infections pose a significant risk to human lives worldwide as well as a significant economic downturn, particularly in countries with limited therapeutic resources. In this study we reviewed the latest proposed technologies that were used to control the impact of respiratory diseases. Artificial Intelligence (AI) is a promising technology that aids in data analysis and prediction of results, thereby ensuring people’s well-being. We conveyed that the cough symptom can be reliably used by AI algorithms to detect and diagnose different types of known diseases including pneumonia, pulmonary edema, asthma, tuberculosis (TB), COVID19, pertussis, and other respiratory diseases. We also identified different techniques that produced the best results for diagnosing respiratory disease using cough samples. This study presents the most recent challenges, solutions, and opportunities in respiratory disease detection and diagnosis, allowing practitioners and researchers to develop better techniques.

## I. Introduction

Lung malfunctioning poses an increased mortality and morbidity risk on the global population. The risk is elevated in developing counties that experience increased pollution due to many factories and lack of efficient air ventilation solutions. Many diseases like asthma, bronchitis, pertussis and COVID-19 share coughing as a common symptom. The cough sound tends to be unique for each respiratory disease enabling physicians to diagnose the illness from the cough sound itself. Therefore, many digital technology solutions that employed big data analysis, Internet of Things (IoT), Blockchain, artificial intelligence (AI) in machine learning (ML) and deep learning (DL), etc. were proposed to identify the disease from the cough sound [1]. Moreover, the healthcare system is engaging more with AI to help doctors in predicting and diagnosing a variety of diseases [2], especially in the past year when the COVID-19 virus became a pandemic and there were not enough hospitals to provide a proper service to the patients [3]. Due to the fatal consequences of respiratory diseases, it is important to develop cost effective and convenient technologies to control them. According to the World Health Organization (WHO), healthcare technologies manifest great contribution in improving treatment of several respiratory disorders. Also, AI is the most promising technology that if employed properly would be so effective in changing the history of disease diagnosis and detection [4].

Several review papers performed a feasibility study on using technology in illness control and management such as [5] that surveyed a range of studies about cost effective disease diagnosing and controlling tools from 55 papers. They stated inexpensive devices like mobile apps, text messaging/SMS and wearable technologies had proved their feasibility in diagnosing different respiratory diseases. Also, Amrulloh *et al.* [6] reviewed the AI techniques that were used to detect asthma disease. The survey discussed the most used AI methods to detect asthma and the most used techniques are ANN (Artificial Neural Network), DT (Decision Tree) and RF (Random Forest). Similarly, Anand *et al.* [7] reviewed the latest technologies that were employed to defeat COVID-19 on different scales. The survey stated how technology helped the medical practitioners to track COVID-19 areas of infection, image processing, and recognizing the best medicine based on the data analysis performed on the patients. Moreover, Bales *et al.* [8] had reviewed the four stages COVID-19 and stated that the economic impact of the pandemic such as decreasing number of using transportation due to the lockdown, a dropdown of the tourism industry unlike food and telecommunication industries that were increased in the pandemic. Furthermore, Belkacem *et al.* [9] discussed the AI and big data suggested solution to overcome the COVID-19 pandemic and offered an outline of the healthcare technological tools for better understanding. They declared that the most used technologies for image scanning are X-ray images and computed tomography (CT) for its low-cost.

Shuja *et al.* [10] had surveyed the most relevant papers that employed artificial intelligence for analyzing COVID-19 from CT-scan image, cough sound and x-ray to create a database for diagnosing and preventing the disease. Also, their research compared the relevant work and set challenges and future direction in the field. They recommended that most of the health providers use technology like image scanning and cough sound analysis via application to protect the forefront workers and find a way to protect the privacy of the patients who shared their data.

Deshpande and Schuller [11] overviewed several techniques of ML/DL used for audio analysis to detect COVID-19 disease. They summarized the COVID-19 diagnostic methods and divided them to cough detection methods, breathing analysis, chest x-ray, and popular chat-pots to automate the communication with physiologists for critical disease analysis. They also discussed the latest monitoring actions done by the governments to control the disease and help doctors to provide the service while keeping the norms of social distancing. Likewise, Lella and Alphonse [12] reviewed the latest technique that used sound analysis to recognize COVID-19 from different respiratory sounds like cough, breathing and voice. It stated that AI methods are reliable and efficient to diagnose COVID-19 and recommended more research work for AI application in this field. They recommended Convolutional Neural Network (CNN) for data crowdsourcing and Data De-noising Auto Encoder (DDAE) to create effective COVID-19 respiratory sound diagnostic tool.

Most of the surveys discussed AI-based detection and diagnostic methods based on various vital signs, but none of them specifically discussed the AI-based cough sound detection method. Furthermore, the field’s challenges and opportunities in this specific area must be updated, because cough detection and diagnosis software is inexpensive and can improve health practices. Motivated by this need, we conducted a survey paper to address the limitations of the previous papers. This scoping survey paper makes the following key contributions:

• Provide characteristics of the diseases and their symptoms.
• Summarize the latest approaches to diagnose cough sound and identify the related respiratory disease.
• Conduct a table that compares the various diagnosing approaches, including their sensitivity, specificity, F1-score, and accuracy.
• Review the most recent methods for detecting cough sounds and diagnosing lung disease.
• Highlight the challenges and opportunities in the field.

The paper is structured as follows: Section 2 illustrates the methodology, then Section 3 provides several cough characteristics and symptoms. Section 4 provides different cough detection and diagnosis methods. Section 5 discusses the most accurate and preferred AI algorithms used in the most related papers, as well as the challenges and opportunities in the cough-based detection and diagnostic industry and market. Lastly, the paper’s conclusion is in Section 6.

## II. Methodology

This paper aims to summarize the proposed approaches that utilized AI/ML to diagnose and detect different respiratory infections through cough sound. Therefore, PRISMA (Preferred Reporting Items for Systematic Reviews and Meta-Analyses) method was followed to search and select the studies related to the scope of the study.

### A. Identification

We first selected the databases from the best-reputed journals and conferences such as IEEE, ACM, and Elsevier that were published between (2012-2021). Different keywords were used to search for the relevant articles, namely cough sound, diagnosis, artificial intelligence, machine learning, respiratory diseases, asthma diagnosis, pulmonary diagnosis, TB diagnosis, bronchitis diagnosis, pertussis diagnosis, and COVID-19 diagnosis. Initially, 93 papers were found, which proposed different AI-based solutions to classify and identify different respiratory diseases using cough sounds. There were no duplicate studies.

### B. Screening and Eligibility

The title and the abstracts of the retrieved papers were initially screened to select the papers based on diagnosis by cough sound. Thus, we found the most relevant 48 papers with regards to respiratory disorder type, dataset size, dataset nature and algorithm performance. All 48 papers were included in the review.

**FIGURE 1.**
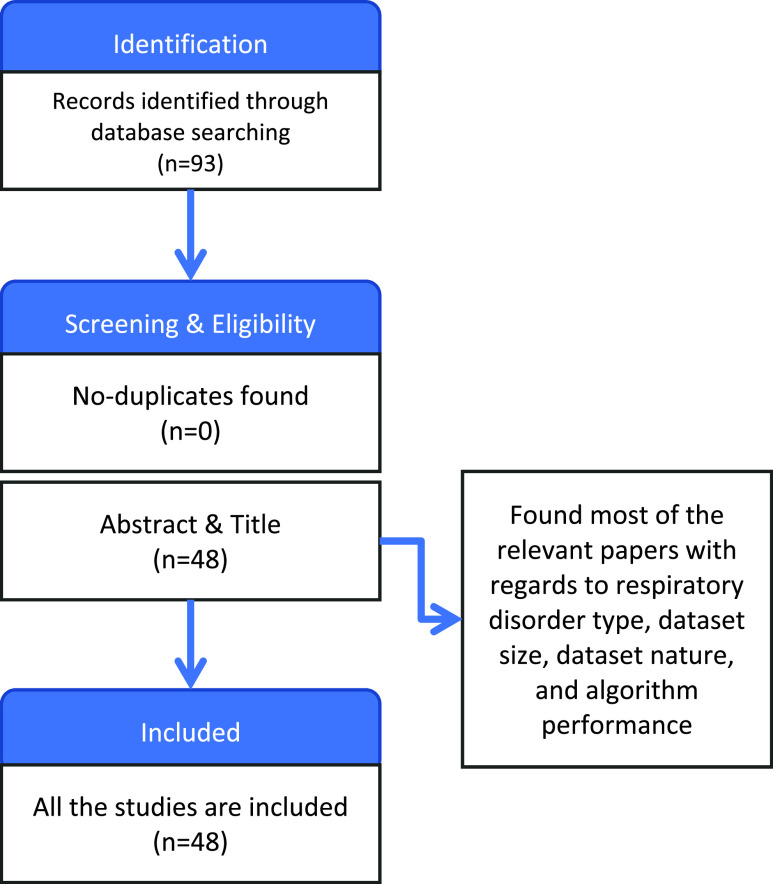
Methodology of the proposed study.

As an outcome of this study, we discussed cough sound detection and diagnosis of different respiratory diseases. We then conducted analysis tables and charts to clarify them as shown in Figure 1.

## III. Characteristics and Symptoms of Several Respiratory Disorders

Knowing respiratory infection symptoms is extremely important to help doctors and technology developers build efficient and effective tools to identify the disease type. In the following subsections we present different respiratory disorders and their related symptoms along with a comparison between them.

Firstly, pneumonia is a respiratory disease that affects children and elderly people above the age of 50 more than young people. It is also more prevalent in men rather than women. It has some symptoms that vary among people such as acute cough, hard breath, crackles, fast breathing, tachycardia and fever [13]. The children can suffer from rib (chest) tightness, difficulty in breathing, wheezing/snoring, flu/running nose, diarrhea, vomiting and fever. Both asthma and pneumonia have chest tightness as a symptom [14].

Secondly, pulmonary disease is a rare chronic respiratory disease that is caused by the spread of small pulmonary arteries, and if not treated it can cause heart misfunctioning and death [15], [16]. It is a serious infection that has many fatal symptoms such as shortness of breath, fatigue, depression, swelling (in ankles/legs/hands), irregular heartbeat, chest illness, headache, fainting and dry cough [17].

Thirdly, huge number of deaths were caused in Indonesia due to tuberculosis (TB) and is the fifth highest death rate in the world [18]. It is diagnosed by rapid nucleic acid–amplification test to examine the genetic formulation of the protein of the virus. Its symptoms include chronic cough, night sweat, fever, weight loss and bloody cough due to the inflammation and destruction of the airways [19].

Fourthly, asthma is an obstructive lung infection that causes many implications for its patients. The wheezing cough is its main symptom followed by others like hyperventilation of the lungs, breathlessness, asthma attack, change birth weight and fever [20]. Also, other fatigues relied on limitations in day-to-day activities, chest tightness and dizziness [21], [22].

Fifthly, bronchitis which is related to obstructive pulmonary disease (COPD) is where the cough and mucus are the main symptoms. It is a very serious illness when left untreated can cause death due to or airflow limitation [23].

Sixthly, pertussis is a respiratory infection that is diagnosed through polymerase chain reaction and serology. It consists of three stages: catarrhal, paroxysmal and convalescent. Its symptoms include whooping sound, fever, cough, cyanosis, red face, swelling eyes and vomiting [24], [25].

Lastly, COVID-19 is an acute respiratory infection with clinical features between acute respiratory distress syndrome (ARDS) and multiple organ dysfunction syndromes (MODS). Its symptoms include fever, dry cough, exhaustion, phlegm, sore throat, lightheadedness, myalgia and hard breath, while symptoms such as nausea, congestion of nose, bloody cough, diarrhea and conjunctival congestion were rarely noticed. Also, if left untreated in elders who are smokers with lung or heart problems, it leads to serious complications like bluish lips/face, ARDS, acute heart injury and secondary infection. The highly infected people by this disease are the elders above 60, or with a medical condition such as diabetes, high blood pressure, asthma and heart disease [26].

Therefore, researchers have to analyze the reasons and the needed technologies to improve the prevention and control of the disease. Table 1 shows the common symptoms for the respiratory diseases according to the studies cited. Furthermore, it is not mandatory that all the patients have all the symptoms. Association of the symptoms with the disease in the table reflect that the selected feature is one of the symptoms that is identified as significant in the studies related to AI-based diagnosis of respiratory diseases.

**TABLE 1:** Symptoms of Different Respiratory Diseases

	Symptoms						
Disease	Pneumonia [80], [49]	Pulmonary [46]	Tuberculosis [11]	Asthma [74], [39], [25]	Bronchitis [43]	Pertussis [16], [19]	COVID19 [63]
Fever	•		•	•		•	•
Dry or bloody Cough		•	•		•	•	•
Breathlessness	•	•		•			•
Sore throat							•
Rib (chest) tightness	•	•		•			
Wheezing/snoring	•			•			
Running nose	•						
Diarrhea	•						•
Vomiting	•					•	
Impairment of lung function			•	•	•		•
Whooping cough						•	
Phlegm					•		
Nausea/myalgia							•
Heart Problems	•	•			•		•
Bluish/red lips/face						•	•
Cyanosis						•	
Hyperventilation of the lungs				•			
Change weight			•	•			
Dizziness/lightheadedness		•		•			•
Depression		•					
Swelling (in ankles/legs/hands)		•					
Night sweat			•				

## IV. Respiratory Disorders Diagnosis and Detection

**FIGURE 2.**
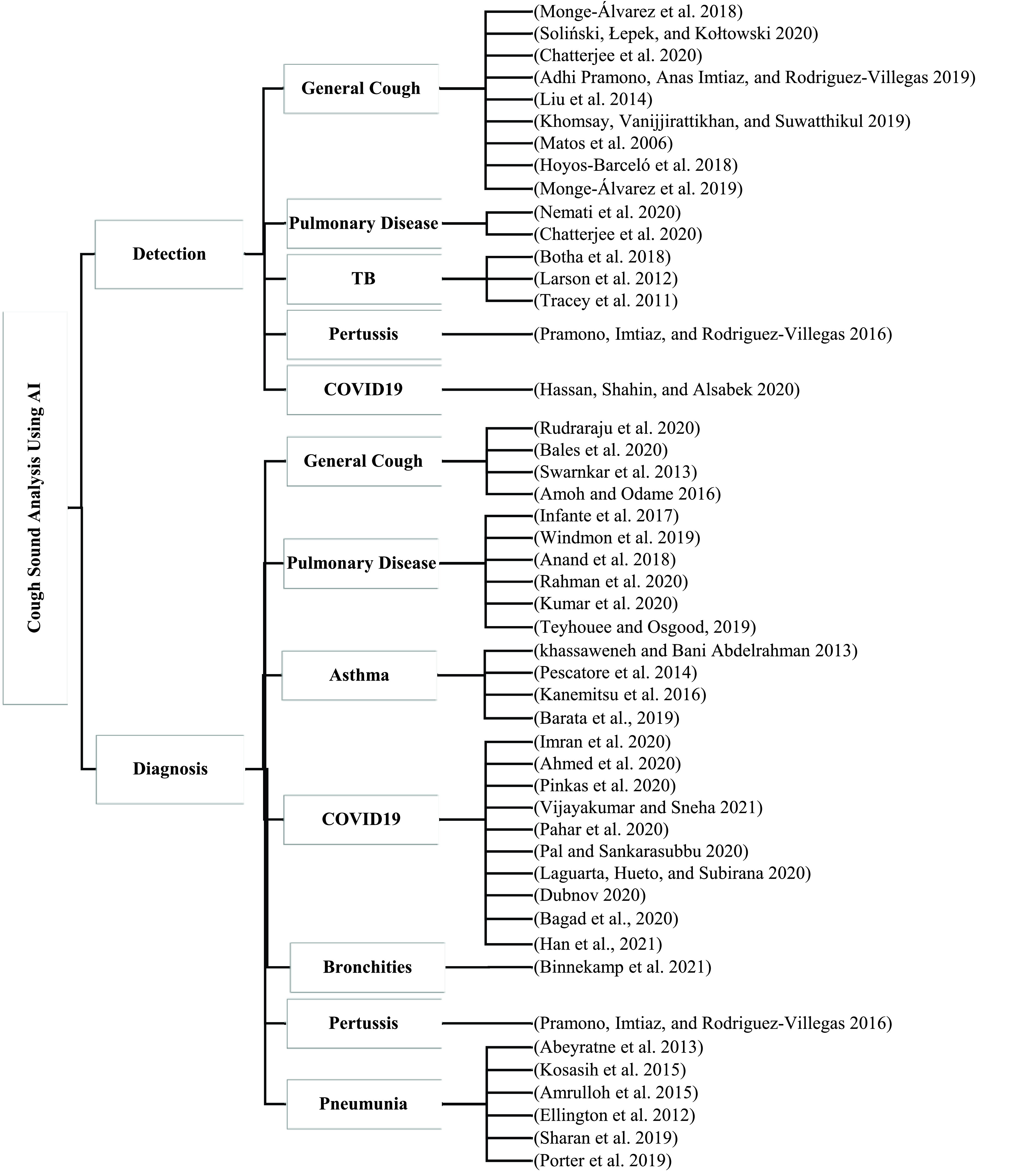
Classification of current cough-based detection and diagnostic methods.

Cough sound detection and diagnosis should be convenient and cost-efficient, with high accuracy and efficiency to be valuable to patients, and doctors, especially in the poorer countries. This review focused on the proposed approaches that used cough sound as an easy detection and diagnosis method to be implemented in affordable and popular platforms such as mobiles, and recorders. So, the best technique to perform both tasks are AI and ML approaches that contain many data analysis and classifications such as Artificial Neural Networks (ANN), Deep Neural Network (DNN), and Recurrent Neural Network (RNN). Also, the classifiers used in AI techniques such as Support Vector Machine (SVM), Convolutional Neural Network (CNN), Mel Frequency Cepstral Coefficient (MFCC), and Hidden Markov Model (HMM). In the following subsections, we discuss approaches that have utilized AI/ML techniques to diagnose and detect the cough sound. Figure 2 shows the classification of studies for respiratory diseases diagnosis using cough samples.

### A. Cough Sound Detection Approaches

Human sounds can vary among normal breath, speaking, cough, whooping, wheezing, snoring, sneezing, and crackling. Detecting cough sounds can be challenging and cause many problems for inexperienced physicians through tools and for that reason advanced automated system monitoring tools are essential. The developed tools need to focus on increasing the accuracy of the predictive algorithm to detect the specific sound despite a noisy background.

Many AI-based approaches and deep learning models were proposed to detect coughs and distinguish respiratory diseases from each other in recording devices and are collected and discussed in these studies. Iyawa *et al.* [27] proposed a mechanism to detect cough sounds in a noisy environment through utilizing MFCC that defines time-frequency decomposition and application of a filter. The proposed system defines the energy pattern for time frames and characterizes frequency bands by using moment theory. It achieved a sensitivity and specificity of around 90%. Also, Kanemitsu *et al.* [28] offered an algorithm to detect cough in spectral sound events by utilizing only three spectral features with LR to separate sound segments into two classifications cough or not cough. The algorithm attained great specificity of 98.14%, sensitivity of 90.31%, and an F1-score of 88.70%. Khomsay *et al.* [29] recommended a hybrid model consists of an ANN model for the preliminary classification and an HMM for the main classification to differentiate between a cough and other types of sounds. The suggested system enhanced detection rates on small SNR from 5 dB down to −10 dB. It detected more than 80% of cough sounds at 5 dB with a constant error rate of around 5%.

Likewise, Kanemitsu *et al.* [28] proposed a technique that applied LR to classify and distinguish cough sound from other sound events. It attained a sensitivity of 86.78%, a specificity of 99.42%, and an F1-score of 88.74%. Kim and Gyoonhee Han [30] had offered a hybrid model based on DNN, and a HMM for cough sound detection. The proposed method was compared to the Gaussian Mixture Model (GMM) and HMM. The suggested model attained a sensitivity of 90.1%, specificity 88.6%, and F1-score 88.6%. Similarly, Kline *et al.* [31] proposed a method to detect cough sounds based on Principal Component Analysis (PCA) to extract features from cough samples, TensorFlow for computation of the features, and Deep Learning Networks (DLN) for cough sound classification. The recording system was built on Raspberry Pi and a microphone recording with 8 volunteers divided based on their ages and genders. The accuracy of PCA+DLN was nearly 99.91% whereas DLN only was about 98.45%.

Furthermore, Kosasih *et al.* [32] proposed a cough detection method to analyze the sound of coughs in spirometry device by sending the sound to the mobile phone that contains the AI models to analyze the cough. The approach used several classifiers, such as LR, ANN, SVM, and RF to pick best performance classifying technique. The ANN achieved 86% sensitivity, 91% specificity, 91% accuracy, and 88% F1-score. Finally, Kumar *et al.* [33] presented a cough detection system for microphones, which used HMMs to denote time-changing features of the cough that is followed by a keyword-spotting approach for the classified coughs. It achieved a high detection rate of around 82% at a false alarm rate of seven incidents/hour, including the incidents exceeding the threshold energy.

Likewise, Monge-Álvarez *et al.* [34] designed a cough detection system by engaging SVM second order polynomial kernel for the classification step to fetch the cough sound from the noisy backgrounds. Their dataset was composed of thirteen patients with different cough sounds and symptoms. Their system attained 92.71% sensitivity, 88.58% specificity, and 90.69% Area Under Receiver Operating Characteristic (ROC) curve (AUC), outperforming state-of-the-art methods.

Other approaches suggested mobile applications for cough sounds detection like Laguarta *et al.* [35] that offered a mobile application to detect respiratory infection by analyzing image moments over audio spectrograms to feed an optimized classifier for final cough detection. The approach implemented Hu moments to increase the functionality of the system by lowering the power usage under 25% or 16% with the consideration of the detector through 24 hours use. It attained 98.64% specificity and 88.94% sensitivity in loud locations. Similarly, Lai *et al.* [36] created a mobile application that detected cough and snoring sounds from a diversity of subjects by utilizing the k-nearest neighbor (k-NN). Their proposed approach consisted of automated ground-truth labeling for energy threshold-based segmentation to distinguish the cough from snoring events. k-NN was used to classify data. Their method achieved an 88% F1-score at signal to noise ratio (SNR) levels of −40dB to 40dB.

There are several approaches in pulmonary disease and TB cough sound detection such as Larson *et al.* [37] approach who created a cough recognition system to distinguish user cough samples from other surrounding subjects’ false coughs. It utilized a neural network and GMM to detect user cough, verify the person, and enhance the system accuracy. It consisted of 5380 cough samples and it achieved 96.37% specificity, 86.55% sensitivity, and 91.46% accuracy. Also, Lewnard and Lo [38] built a smartphone app to assess and detect a pulmonary disease from wheeze sound. The study was conducted on 131 participants between healthy and ill subjects. It utilized RF with 1000 trees, Adaboost, and Gradient-Boosted Tree (GBT) to detect the wheezing sound from the patient. Their method achieved 84.14% specificity, 74.62% sensitivity, 94.6% accuracy, and 79.47% F1-score. Also, Cornia and Lipsky [19] offered a cough detection system from the audio recording by a smartphone or tablet where they utilized HMM and MFCC to extract specific features from the cough. Their detection method consisted of cough samples for diagnosis, although they recommended conducting many samples such as sputum to better diagnose Tuberculosis (TB). Also, Liu *et al.* [39] and Lytras *et al.* [40] utilized DNN and SVM to detect TB cough from the noisy environment by requesting the subject to input their cough in the enrollment phase. Lytras *et al.* [40] used Multilayer Perceptron Model (MLP) to achieve 88.2% accuracy on 13,429 cough frames and 43,925 non-cough events and divide-and-conquer? clustering algorithm to update the data regularly when the newer set is available. The Liu *et al.* [39] method using sequential minimal optimization (SMO) to correctly detected 75.5% of cough episodes and achieved higher specificity 99.6% on the same sample size. Similarly, Maghded *et al.* [41] employed DNN to detect TB infected patients from lung color photo to be identified by an algorithm stored in the Android mobile camera. Their method utilized RF algorithm and achieved 98.4% accuracy.

Recently, many researchers improved mechanisms to detect COVID-19 in patients. For example, Matos *et al.* [42] utilized Recurrent Neural Network (RNN) and the Long Short-Term Memory (LSTM) to detect and assess the characteristics of COVID19 cough and other respiratory sounds. They employed LSTM to save data permanently in the memory cells to retrieve the old data. Their method achieved 97% accuracy, 97.9% F1-score, Recall 96.4%, Precision 99.3%, and AUC 97.4%. Another study for COVID-19 detection proposed by McCollister *et al.* [43] created mobile-based and web-based surveys to identify COVID-19 cases fast through ML. They stated that the methods helped to reduce the virus spread among quarantined people. The proposed algorithm identified COVID-19 infected people with minor symptoms. Table 2 shows the summary of studies related to general cough detection.

**TABLE 2:** Comparison of General Cough Detection Approaches

Ref	Disease	Method	Dataset	Size of the data	Specificity	Sensitivity	F1-score	Accuracy
[48]	Cough and non-cough (e.g., asthma, bronchiectasis, or chronic obstructive pulmonary disease)	Mel frequency cepstral coefficients (MFCC)/moment theory	Cough sound	–	90%	90%	–	–
[69]	Cough and non-cough	Artificial neural network (ANN)	Cough sound	19,832 Sound	91%	86%	88%	91%
[2]	Cough and non-cough	Logistic regression	Cough sound	1980 Sounds	99.42%	90.31%	88.74%	–
[39]	Cough and non-cough	Deep neural networks (DNN), and hidden Markov model (HMM)	Cough sound	45000 Sounds	88.6%	90.1%	88.6%	–
[29]	Cough and non-cough	Principal Component Analysis (PCA), and Deep Neural Networks (DNN)	Cough sound	810 Events	–	–	–	99.91%
[42]	Cough and non-cough	HMMs	Cough sound	–	–	–	–	82%
[34]	Cough and non-cough	SVM	Cough sound	13 cough	88.58%	92.71%	–	90.69%
[88]	Cough and non-cough	Hu moments	Cough sound	–	98.64%	88.94%	–	–
[8]	Cough and non-cough	Convolutional Neural Networks (CNNs)						
[77]	Cough and snoring	K-nearest neighbor (k-NN).	Cough sound	26 Subjects	–	–	88%	–
[58]	Pertussis	MFCC	Questionnaire, Cough sound	414 Coughs	90%	92.38%	–	–
[46]	Pulmonary disease or asthma	Cough embeddings Cosine – cough detection	Cough sound	5380 Cough samples	96.37%	86.55%	–	91.46%
[18]	Pulmonary disease	Random Forest with 1000 trees (RF_1000), Adaboost, and Gradient-Boosted Tree (GBT), root-mean-square energy cough detection	Cough sound	8,491 cough samples	84.14%	74.62%	79.47%	94.6%
[14]	Tuberculosis	HMM and MFCC	Cough sound	746 Coughs	72%	95%	–	78%
[37]	Tuberculosis	SVM, DNN, sequential minimal optimization (SMO)	Cough sound	13,429 Cough frames and 43,925 non-cough frames	99.6%	75.5%	–	–
[76]	Tuberculosis	DNN, MLP, SVM	Cough sound	13,429 Cough frames and 43,925 non-cough frames	–	–	–	88.2%
[87]	COVID-19	Recurrent Neural Network (RNN) and the Long Short-Term Memory (LSTM)	Cough sound	–	–	–	97.9%	97%

### B. Cough Diagnosis Approaches

This section introduces several cough-based diagnoses approaches and is organized as follows. Subsection (1) provides general cough diagnosis approaches. While subsection (2) discusses pneumonia diagnosis approaches. Subsection (3) introduces asthma and pulmonary disease diagnosis approaches. Finally, subsection (4) provides COVID19 diagnosis approaches.

#### 1) General Cough Diagnosis Approaches

Cough diagnosis is important to help practitioners identify the type of respiratory disease. For example, whooping sound is one of the main symptoms of pertussis respiratory disease [44]. There is an approach that was conducted by Morrell *et al.* [45] to diagnose pertussis from the cough sounds and classifying the whooping sound in pertussis patients through using Logistic Regression Classifier (LR). The method worked on mobile phones and did not require a person to record the sound for tuning threshold. It accomplished 92.38% sensitivity and 90% specificity.

The main limitation of this work was the absence of testing the power consumption of the algorithm in small devices although they stated that it performed better in large memory and power devices. Another study by Nemati *et al.* [46] utilized Multiplex Ligation-dependent Probe Amplification (MLPA), Polymerase Chain Reaction (PCR) to diagnose the sound of bronchitis coughs among trained pediatricians. Their method tested the accuracy of this kind of test in identifying the cough type and the feasibility of the questionnaire for the practitioners. It included 16 cough sounds to be tested and it achieved 76.2% sensitivity and specificity. Also, Nemati *et al.* [47] proposed a ML model that combined standard signal processing features and domain-specific features that aided in differentiating sound of the cough collected by the spirometry. It attained 92.38% sensitivity and 90% specificity.

The following AI techniques were proposed to diagnose cough sounds and differentiate between them to provide accurate treatment. Monge-Álvarez *et al.* [48] utilized Convolutional Neural Networks (CNNs) to distinguish cough sounds in an audible environment to diagnose and differentiate between three types of diseases, bronchitis, bronchiolitis, and pertussis. The proposed model achieved 89% accuracy. Also, Ozkaya *et al.* [49] recommended a method to recognize cough sounds based on their condition either wet or dry by employing logistic regression (LR). Their method specified the person based on his/her gender and age. It achieved 84% sensitivity and 76% specificity. Moreover, a deep learning model was suggested by Pahar *et al.* [50] that utilized deep neural network (DNN) by amalgamating a convolutional neural network (CNN) and a recurrent neural network (RNN) to distinguish cough from other sounds. In the proposed approach, they made a comparison of performance between CNN and RNN which showed that the CNN yields an overall higher accuracy of 89.7%. The CNN-RNN approach achieved a specificity of 92.7% along with a sensitivity of 87.7%. Table 3 presents the summary and comparison of general cough diagnosis approaches.

**TABLE 3:** Comparison of General Cough Diagnosis Approaches

Ref	Disease	Method	Dataset	Size of the data	Specificity	Sensitivity	Accuracy
[58]	Pertussis	LR	Questionnaire, Cough sound	414 Coughs	90%	92.38%	–
[13]	Bronchitis	Multiplex ligation-dependent probe amplification (MLPA), and PCR Polymerase chain reaction	Questionnaire, cough sound	16 Cough	76.2%	76.2%	–
[60]	Cough (e.g., asthma, bronchiectasis, or chronic obstructive pulmonary disease)	MFCC	Cough sound	1700 coughs	93.69%	87.2%	91.97%
[8]	Cough (e.g., asthma, bronchiectasis, or chronic obstructive pulmonary disease)	CNNs, MFCC	Cough sound	268 Coughs	–	–	89%
[75]	Wet or dry cough diagnosis	LR	Cough sound	310 Cough	76%	84%	–
[5]	Cough (e.g., asthma, bronchiectasis, or chronic obstructive pulmonary disease)	DNN, CNN, and RNN	Cough sound	627 Coughs	92.7%	87.7%	–

#### 2) Pneumonia Diagnosis Approaches

Pneumonia is an acute lower respiratory infection (ALRI) and it affects more than 1.6 million children around the world. The detection of the disease may include chest radiography, clinical diagnosis, physical exam, imaging, oxygen saturation measurements, and lung ultrasound [51]. Cross wavelet transform (CWT) can also be utilized to analyze cough sound signals for pneumonia diagnosis. It differentiates between a crackled signal which has a frequency less than 1000 Hz and the pneumonic cough sound duration lasts for 400/450 ms with the presence of crackle in Pescatore *et al.* [52]. Another study by Pham *et al.* [53] utilized a wavelet-based sound detection method to diagnose pneumonia cough from other respiratory coughs like asthma and bronchitis. Their model combined extracted sound feature with MFCC and non-Gaussianity index for high sensitivity and accuracy. It achieved sensitivity and specificity of 94% and 63%, respectively, and the best accuracy of 84% by using Morlet and Du wavelets.

Asthma symptoms overlap with pneumonia, but different treatment is required with a very precise diagnosing tool to be able to differentiate between the two infections [54]. Therefore, Pal and Sankarasubbu [51] proposed an alternative to electronic auscultation and chest ultrasonography for lung sound detection and analysis. Their technique consisted of a cough-diagnosing method to separate pneumonia from asthma by utilizing AI techniques such as KNN methods, SVM, RF, and GB along with a questionnaire to the patients. Their method achieved 80% specificity.

Similarly, Pingale and Patil [54] offered a cough sound diagnosing technique to distinguish between asthma and pneumonia through AI. It utilized the HMM classifier to recognize pneumonic coughs and asthmatic coughs. Their study was conducted on 20 participants half of which were pneumonic and the other half asthmatic, and it achieved 90% accuracy, 100% sensitivity, and 80% specificity.

Several approaches utilized MFCC for characteristics classifications such as Pinkas *et al.* [55] who proposed a pneumonia cough diagnosing system for microphones by employing non-Gaussian, MFCC, and Logistic Regression (LR) for sound classification. It resulted in a high detection specificity of 75% and sensitivity of 94% based on the parameters of extraction. Also, Porter *et al.* [56] proposed an automated cough sound analysis technique to diagnose cough sounds for different diseases using MFCC on mobile phones. The approach achieved high accuracy in detecting respiratory diseases as follows: asthma (97, 91%), pneumonia (87, 85%), lower respiratory tract disease (83, 82%), croup (85, 82%), and bronchiolitis (84, 81%). Also, Pramono *et al.* [57] presented a croup diagnosis method to discover pneumonia in the patients. They used SVM and MFCC for data classification and it achieved a sensitivity of 95.24% and specificity of 90.00%. Table 4 presents the comparison of pneumonia cough diagnosis.

**TABLE 4:** Comparison of Pneumonia Cough Diagnosis

Ref	Disease	Method	Dataset	Size of the data	Specificity	Sensitivity	Accuracy
[1]	Pneumonia	MFCC and LR	Cough sounds and vital signs	16 Coughs	75%	94%	–
[32]	Pneumonia	Wavelet-based crackle detection/Morlet and Du wavelets	Cough sounds	815 Coughs	88%	94%	84%
[6]	Asthma and pneumonia	HMM	Cough sounds	461 coughs from pneumonia 277 coughs from asthma	80%	100%	90%
[24]	Pneumonia	k-NN, SVM, RF, GB and MFCC	Cough sounds and questionnaire	Lung sounds from 600 children	80%	–	–
[63]	Pneumonia	MFCC and SVM	Cough sounds	364 Coughs	85.29%	92.31%	–
[56]	Pneumonia	MFCC	Cough sounds and questionnaire	500 Coughs	–	–	87, 85%

#### 3) Asthma and Pulmonary Disease Diagnosis Approaches

Asthma is a serious respiratory disease that causes severe injuries to human lungs. It is related to pulmonary disease. Pramono *et al.* [58] proposed an asthmatic cough sound diagnosis technique from a recorder by utilizing signal processing technique known by Wigner distribution function in a noise-free room. Their method used MATLAB to run the classifier which classifies a cough as either asthmatic and non-asthmatic along with the gender and age properties. However, we cannot consider this method at the same level as other diagnostic methods since the size of the dataset is small.

Many studies proposed mobile apps to diagnose and detect asthma and pulmonary disease such as Rahman *et al.* [59] which proposed a mobile application that detects wet and dry coughs from pulmonary disease patients by operating wrapper-based feature selection algorithms. Their proposed approach attained 86% sensitivity and 84% specificity. Another approach proposed by Rahman *et al.* [59] adopted a cough-diagnosing method through an embedded sensor in the patient’s mobile. The approach achieved 90% sensitivity and 76% specificity. They mentioned that their proposed diagnosis device is more accurate and convenient than spirometry.

Rudraraju *et al.* [60] suggested a technique to diagnose asthma disease by utilizing a Gaussian Mixture Model–Universal Background Model (GMM-UBM). The data was collected through a questionnaire that collected information on demographical data, cough duration, and patient respiratory history. Their method achieved 82.81% sensitivity, 84.76% specificity, and 80% overall accuracy in diagnosing coughs in the absence of wheezing sounds. Also, Rusdah and Wardoyo [61] designed a prognostic model to diagnose children infected with asthma through their wheezing sounds or coughs. The study utilized LASSO-penalized logistic regression for model training, and it achieved 71% specificity and 72% sensitivity in cough diagnosing. Another study proposed by Shabut *et al.* [62] developed a mobile phone application called TussisWatch to record cough sounds and diagnose different diseases such as chronic obstructive pulmonary disease (COPD) and congestive heart failure (CHF). The approach utilized a random forest classifier to process cough sound and it achieved an accuracy of 80.67%, specificity of 82%, and a sensitivity of 80%. Fractional exhaled nitric oxide (FeNO) measurement was another approach employed by Sharan *et al.* [63] along with an airway responsiveness test to diagnose cough variant asthma (CVA). The FENO measurement is set to 22 ppb or higher for asthma cough, so any lower value will not be considered as an asthma disease.

Barata *et al.* [64] utilized convolutional neural networks to create a classifier to reduce cross-device discrepancy for asthmatic cough sound detection in mobile phones. They applied their system on 43 subjects and recorded 6737 cough samples and 8854 control sounds by 5 different recording devices and they achieved 85.9%, 90.9% accuracies. They suggested to increase the quality of recorded cough sound to enhance the quality of the classification.

A variety of studies focused on diagnosing pulmonary disease like Shi *et al.* [65] who designed a DL model for pulmonary cough sound detection via microphone and stethoscope for vital signs. It consisted of a handheld sensor questionnaire for collecting the vital signs and a website to store the patient’s data. It achieved 18% sensitivity, 67% F-score, and 41% accuracy. Also, Shin *et al.* [66] proposed a pulmonary disease diagnostic tool. Cough samples were collected using mobile phones, while questionnaires were used to collect other data such as vital signs. Their method attained 81% specificity, 81% sensitivity, an overall 81% accuracy. Likewise, Small *et al.* [67] offered a mobile toolkit called “pulmonary screener” for cough sound diagnosis to distinguish among popular pulmonary infections (Asthma, COPD, and allergic rhinitis (AR)). It utilized ML to calculate the statistical analysis for the disease possibility and it achieved 90% accuracy, specificity, and sensitivity in all respiratory diseases except asthma which achieved 84% accuracy. Also, Teyhouee and Osgood [68] proposed a machine learning model by utilizing multiadaptive HMM to classify pulmonary cough sounds from other environmental noisy sounds based on energy band and time series. Their method achieved 92% AUC. Table 5 shows the comparison of pulmonary disease and asthma cough diagnosis.

**TABLE 5:** Comparison of Pulmonary Disease and Asthma Cough Diagnosis

Ref	Disease	Method	Dataset	Size of the data	Specificity	Sensitivity	F1-score	Accuracy
[4]	Asthma	Signal processing techniques	Cough sound	12 asthmatic and 12 healthy male and female	–	–	–	100%
[52]	Asthma	Prediction model (LASSO-penalized logistic regression)	Cough sound, survey and questionnaire	1226 symptomatic children (345 (28%) had asthma)	71%	72%	–	–
[28]	Asthma	FeNO measurements and an airway responsiveness test	Questionnaire	Cold air and talking sounds from 163 patients	81%.	44%	–	–
[64]	Asthma	CNN	Cough sound	6737 cough samples and 8854 control sounds by 5 different recording devices from 43 subjects	–			90.9%
[83]	Pulmonary disease or asthma	LR	Cough sound, questionnaire and vital signs	54 patients (22 healthy)	81%	81%	–	81%
[84]	Pulmonary disease	RF	Cough sound, survey	100 coughs	82%	80%	–	80.67%
[7]	Pulmonary disease or asthma	LR and Bayesian Network (BNN)	Cough sound and questionnaire	325 patients	84%	84%	–	90%
[59]	Pulmonary disease or asthma	–	Cough sound	228 COPD patients	76%	90%	–	–
[85]	Asthma	Gaussian Mixture Model–Universal Background Model (GMM-UBM)	Cough sound, vital signs and questionnaire	1192 patient cough sounds, and 1140 healthy cough sounds	84.76%	82.81%	–	80%
[33]	Pulmonary disease	DNN and confusion matrix	Cough sound, vital signs and questionnaire	108 Subjects		18%	67%	41%
[68]	Pulmonary disease	HMM	Cough sound	–	–	–	–	92%

#### 4) COVID-19 Diagnosis Approaches

A pandemic can be defined as the emergence of a disease that leads to a fast rise in deaths and infection rates on a large-scale [1]. Recently, the COVID-19 pandemic spread rapidly worldwide and caused unprecedented infections and deaths with WHO reporting around 13,616,593 and 585,727 in 216 countries [69], [70]. Several studies proposed the automated diagnosis of COVID-19 using X-ray, CT-Scan, clinical data, vital signs, and cough samples [71], [72], [12], [70], [80].

Srinivasa Rao and Vazquez [73] proposed a cross-correlation adaptive algorithm to synchronize the sound of COVID-19 cough from several devices such as accelerometer and smartwatch. Their proposal solved synchronization issues such as unpredictable clock frequency, thread scheduling, temperature or power. The proposed adaptive algorithm achieved synchronization of 98.9% cough events from various items with a moderate synchronization error of 0.046s.

Many cough diagnosis schemes were proposed utilizing DL models to increase diagnosis accuracy. Svanes *et al.* [74] employed three classifiers Deep Transfer Learning-based Multi-Class classifier (DTL-MC), Classical Machine Learning-based Multi-Class classifier (CML-MC), and Deep Transfer Learning-based Binary Class classifier (DTL-BC) that operate as an ensemble classifier which determines whether or not a cough was produced by a COVID-19-infected person. Their contribution consists of a cloud-based mobile application named AI4COVID-19 that filters the model constantly whenever new data is made available, as there is no need for a specific app to carry the update in the backend of the AI diagnosis engine. DTL-BC has F1-score 92.97%, 94.57% sensitivity, 91.14% specificity, 92.85% precision, and 92.85% accuracy in COVID-19 detection. Although it achieves great results in accuracy, [9] stated that the employment of AI4COVID-19 at a big scale is now restricted by some concerns like dataset size and quality and shortage of clinical tests. Swarnkar *et al.* [75] developed a mobile phone-based ML model to differentiate COVID-19 coughs from healthy coughs. The neural network model used MFCCs for feature extraction and implemented different classifiers to find that ResNet-50 was the best classifier that achieved high precision and area under the curve (AUC) by 98%.

Tracey *et al.* [76] offered a DNN framework to classify cough sounds of COVID-19 from others with four cough classes (COVID-19, asthma, bronchitis, and healthy). They stated that using cough samples along with demographic data and symptoms can increase the accuracy, sensitivity, and specificity. Pinkas *et al.* [55] proposed a mobile application for voice screening to detect SARS-CoV-2 respiratory infections by utilizing deep machine learning and speech processing. The approach contained three stages including a supervised transformer, RNN for sub-models, and ensemble stacking. The approach yielded a recall of 78% and resulted in a probability of false alarm (PFA) of 30%. Furthermore, Vhaduri *et al.* [77] proposed a DL method to diagnose COVID-19 cough and differentiate it from other respiratory diseases. Their proposed method achieved 94% sensitivity.

Vijayakumar and Sneha [78] created a website for cough data recording to build an AI model for cough sound diagnosing. The method extracted sound features and converted them to MFCC and fed them into a CNN that consists of one biomarker layer, and pre-trained ResNet-50s. It achieved a sensitivity of 98.5% and a specificity of 94.2%. van Vugt *et al.* [79] proposed a cough sound diagnostic method to distinguish COVID-19 by utilizing three classifiers which were bicoherence analysis using SVM, Biphase analysis using LR, and MFCC analysis using CNN. The last classifier achieved a higher F-1 score of 70%, and better accuracy with RF 84%, sensitivity with 98%, and 89% specificity with SVM.

Bagad *et al.* [81] created mobile application that collected cough sound from patients to diagnose COVID-19 cough sounds by using AI to speed up the cough diagnosis process. The sample size consisted of 3,621 coughs collected through phone and it increased the testing capacity to 43% at disease prevalence. Also, Han *et al.* [82] created a mobile application with voice-based model and crowdsource data of 828 samples from 343 participants of COVID-19 patients and health people. They used SVM to diagnose COVID-19 by symptoms and voice signals where it achieved AUC of 0.79 with a sensitivity of 0.68 and a specificity of 0.82.

Table 6 shows the summary of COVID-19 diagnosis using cough samples.

**TABLE 6:** Comparison of COVID-19 Cough Diagnosis

Ref	Disease	Method	Dataset	Size of the data	Specificity	Sensitivity	F1-score	Accuracy
[86]	COVID-19	Deep Transfer Learning-based Binary Class classifier (DTL-BC)	X-rays and CT scans of alive COVID-19 patients, cough sound	1838 Cough sounds and 3597 non-coughs	91.14%	94.57%	92.97%	92.85%
[3]	COVID-19	Cross-correlation adaptive algorithm	Cough sound and the movement during cough recording	10000 Coughs	–	–	–	–
[55]	SARS and COVID-19	RNN	Cough sound	5971 Coughs	–	–	–	78%
[78]	COVID-19	SVM	Cough sound	570 Coughs	–	94%	–	–
[50]	COVID-19	LR, SVM, multilayer perceptron (MLP), CNN, LSTM, and a residual-based neural network architecture (ResNet-50)	Cough sound, questionnaire	Sample 1(92 COVID-19 positive and 1079 healthy subjects) Sample 2 (8 COVID-19 positive and 13 COVID-19 negative subjects)	96%	91%	–	92.91%
[51]	COVID-19	DNN	Cough sound	30000 audio segments, 328 cough sounds from 150 patients with	95.04%	90.1%	–	96.83%
[35]	COVID-19	CNN	Cough sound	5,320 Coughs	94.2%	98.5%	–	97%
[21]	COVID-19	CNN	Cough sound	1811 Coughs	89%	98%	70%	84%
[81]	COVID-19	AI	Cough sound	3621 coughs	–	–	–	–
[89]	COVID-19	SVM	Cough sound	828 samples from 343 participants	82%	68%	–	–

## V. Discussion

**FIGURE 3.**
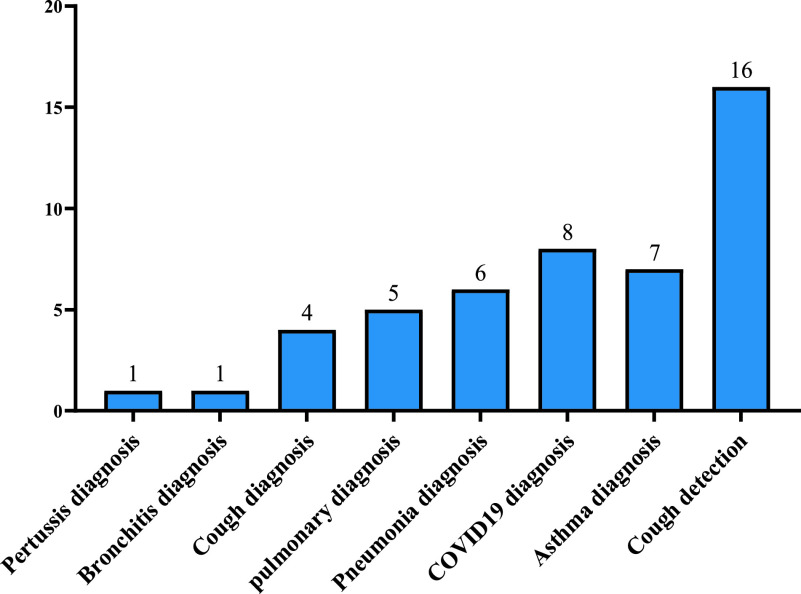
Number of cough detection and diagnosis studies.

**FIGURE 4.**
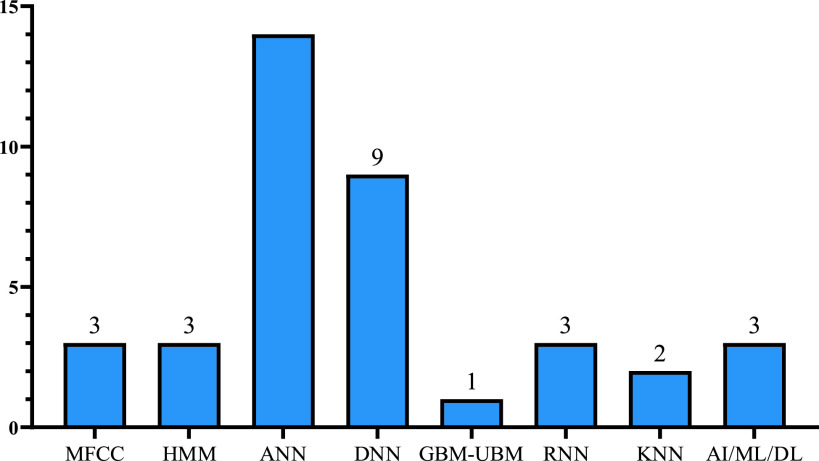
Popular AI/ML techniques used in the current cough-based detection and diagnosis approaches.

**FIGURE 5.**
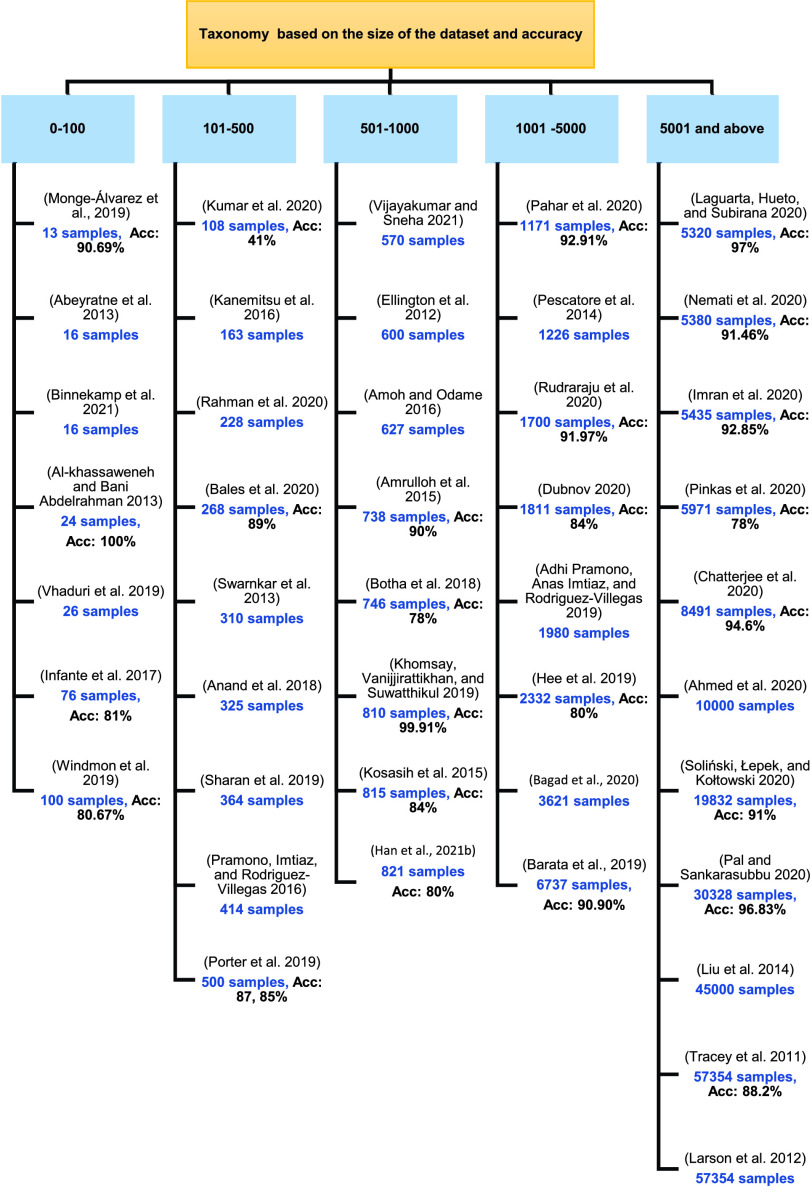
Taxonomy of the state of art dataset size.

From the above tables we identified the widely used technique in detecting and diagnosing cough sound which is the Artificial Neural Network (ANN) and Support Vector Machine (SVM) followed by Logistic Regression (LR) classifier. Deep Neural Network (DNN) is the second-highest method used in diagnosing disease with its classifiers random forest (RF) followed by the convolutional neural network (CNN). There are more algorithms proposed for detection than for diagnosis. Also, other techniques were proposed which are not related to machine learning such as Wavelet-based crackle detection/Morlet and Du wavelets, signal processing techniques, prediction model (LASSO-penalized logistic regression), FeNO measurements and an airway responsiveness test, cough embeddings cosine and PCR but those techniques were used to diagnose and detect respiratory diseases, as shown in the Figures 3, 4 and 5.

As shown in Tables 2, 3, 4, 5 and 6, the datasets used in the cough sound detection and diagnosis approaches reviewed in this paper consist mainly of cough sound samples for a specific respiratory disease. These samples were collected by recording the cough sound for a sample of people (infected and uninfected). However, some studies utilized other types of data including vital signs, and questionnaire data. To improve the performance of the model, it is trained on a large number of samples as shown in Figure 6. The dataset size used in the reviewed studies ranged from 16 to 57,354 samples, noting that the size of the dataset exceeded 500 samples for 24 studies. However, four studies did not report the size of the dataset used.

### A. Challenges and Opportunities in Cough Detection and Diagnosis Technologies

The field of AI and big data is promising in fighting against several respiratory diseases and recently the COVID-19 pandemic. In this section we presented some challenges and opportunities that regulators and developers can use to enhance the effectiveness and quality of the proposed AI-based solutions. van Vugt *et al.* [79] discussed that the core challenge is the heterogeneity of the dataset, the lack of tools to test the feasibility of deep learning models, unbalanced data in the dataset, easily predicted or null data in the dataset, the generality of the machine learning model, a larger number of negative images than positive images which causes an error in prognostication, and the reliability of AI hardware and software. Below we discuss in detail the challenges and chances of improvement shown in the Table 7.

**TABLE 7:** Properties and Challenges in the State of Art Proposed Methods

Ref	Findings	Challenges
[1]	Using fever as parameter for diagnosis increased the sensitivity from 33% to 94%	The study was carried on a small sample size
[32]	Simplicity to detect features of the cough and low-cost implementation	Existence of the crackle is not necessary to detect pneumonia and that led to specificity reduction
[6]	Method uses non-contact measurements; therefore, it does not need extensive sterilization procedures	Increased computational time due to using long windows in the classification
[24]	Improve the accuracy of WHO case management algorithm for pediatric pneumonia.	High cost and limited number of staff to test Not widely tested
[63]	The SVM was determined to be more accurate than LR in classifying the cough sound signals, particularly with the bio-mimicking features	–
[56]	Low-cost solution can be implemented in mobile phones	Noisy sounds for 4.1% of files
[4]	Algorithm compliance with physician diagnosis	Small sample size.
[52]	Clear sound took from children	Interpreted missing values in potential predictor variables as an absence of the respective risk factor, which might also have affected the results. However, the number of missing values did not exceed 5.8% in any of the potential predictor variables.
[28]	“Cold air” and/or “talking” as cough triggers	The exact role of “cold air” and/or “talking” as cough triggers in the pathogenesis of CVA remains unclear. Functional analysis using single photon emission computed tomography or functional magnetic resonance imaging need to be made for validation
[83]	Good detection of different pulmonary diseases	Small sample size.
[84]	Low cost to be implemented on smartwatch	Small sample size, and the low accuracy
[7]	Low-cost mobile app	Unclear classification due to limited features because it is only diagnosing Asthma, COPD, and Allergic Rhinitis, and other related diseases to pulmonary disease are classified under other diseases.
[46]	Low-cost mobile app	Need personal cough in the enrollment phase
[18]	Low-cost mobile app	Noisy sound
[59]	Low-cost mobile app	Small sample size
[85]	Low-cost mobile app	low accuracy
[33]	–	Small sample size
[14]	Convenient, and easy to apply	Small sample size
[37]	Good detection	Slow performance due to huge dataset
[76]	Low-cost system	Slow performance due to huge dataset
[62]	Low-cost mobile app	Noisy image
[25]	Increased accuracy due to utilizing genetic algorithm (GA) and multilayer neural networks (MLNNs)	High time consumption
[55]	Low-cost mobile app	Low performance
[78]	Low-cost solution	Small sample size
[50]	Low cost and can be applied in mobile	No update for the dataset online
[51]	Low-cost	No update for the dataset online
[35]	Low-cost	–
[21]	–	Low performance
[58]	High accuracy	Small dataset size and high complexity system
[13]	Low cost	Small dataset size
[60]	Low cost	Number of features affect the performance and battery consumption
[48]	Achieved overall good cough detection capability and noise robustness	–
[69]	High sensitivity in spirometer	Number of features affect the performance and battery consumption
[2]	High performance	Low number of features in classification
[39]	Good performance	Low quality sound
[8]	Low complexity and good performance	Small dataset size
[75]	Simultaneous implementation with other potential technologies such as microwave imaging and ultrasound imaging that may be capable of detecting consolidations and mucus in lungs	70% of the coughs were dry, so it degrades the classification accuracy
[29]	Good accuracy	Unclear voice samples due to low processing capabilities of the Raspberry Pi
[5]	More specificity due to the hybrid model MFCC + SVM	Slow performance
[42]	Good detection performance	Low energy cough signals producing a lower detection rate
[88]	Low-cost mobile app	Small dataset size, the efficiency is dependent on the dataset size, and no real time update on the database
[77]	Low-cost mobile app	Highly affected by the background noise
	High accuracy	Small dataset size
[64]	Different recording devices system	Low quality sound
[68]	Good detection	Unmentioned sample size
[71]	Good detection	Feature extraction problems
[81]	Low-cost mobile app	No accuracy results mentioned
[89]	Low-cost mobile app	–

### B. Limitations and Solutions

Healthcare technologies occupy a large share in the market because many consumers find them cost-effective, convenient, and easily adoptable [90]. Also, one of the latest concerns worldwide is COVID-19 virus, so scientists used the AI techniques from different respiratory diseases detection and diagnosis to protect against it. This field holds numerous challenges, solutions and opportunities which we discuss in the next sections.

#### 1) Legal Duties for Infectious Disease Control

The outbreak of lung infections highlights the importance of authorities enforcing key countermeasures such as lockdown, social distancing, screening and testing on a large scale. Therefore, it is critical to specify requirements and tasks for residents, scientists and researchers, industry and businesses in order to prevent the disease from spreading. For example, remote diagnosis using AI-based cough sound analysis could be a regulatory requirement for timely diagnosis before traveling or attending large gatherings to limi infection.

#### 2) Low Quality of the Recordings

Background noise and in some cases uncontrolled patients in the environment affect the quality of samples used in the training dataset for detecting and diagnosing Covid-19. It is crucial to figure out what factors, whether environmental or mechanical are causing the sound quality to deteriorate. Then for each of these factors devise a number of solutions [91].

#### 3) Reliability

The reliability of software and hardware is one of the main challenges in AI-based solutions for medical disease diagnosis. The solution to this issue is to design smartphones with enhanced power and sensing capabilities along with a system on chip (SoC).

#### 4) Privacy and Security Issues

Privacy raises a huge concern due to the sensitivity of the patient data that is collected among different low capability storage and power devices [92]–[94]. Also, some patients prefer face-to-face conversations with their physicians, necessitating the need to keep communication channels secure and protected from cyberattacks. This can be addressed by increasing patient trust by enforcing laws and policies that protect patient data with data encryption. Several tools are available to help with security and privacy issues during the pandemic. We have highlighted a few solutions below:

##### a: Blockchain

This technology offers an immutable, distributed database in which all network nodes can verify the transactions using consensus algorithms. Since the blockchain is a reliable solution to enhance the privacy and security of communication, it has been employed in many healthcare platforms [93].

##### b: Privacy-Preserving Incentive Mechanism

Designing an incentive program is critical for enlisting the assistance of a large number of users to contribute to the public database. The purpose of incentive mechanism is to encourage users providing samples to enrich the dataset used for further diagnoses and detection. However, incentive mechanisms must involve both rewarding and privacy protection techniques to convince users to participate [95], [96].

#### 5) Using Mobile Technology for Detection and Diagnosis

The use of mobile devices for detection and surveillance is controversial due to their inability to assess clinical cost and effectiveness. The solution is to enlist the help of reputable third parties to assess the detection system’s effectiveness.

### C. Opportunities

Reviewing the state-of-the-art literature, we found that many applications were created to combat respiratory diseases and improve diagnostic accuracy. Most of the previously proposed models foster opportunities for different sectors including government, citizens, market and developers.

#### 1) Authorities More Involved

By involving authorities in the development of pandemic prevention strategies such as social distancing, lockdown and in-house diagnosis, communication and collaboration among scientists, doctors, developers and business owners improves in order to face the pandemic. They maintain a unified database of the positive cases of any respiratory disease cough sound and symptoms in order to improve diagnosis precision, case prediction and vaccine delivery.

#### 2) Development of New AI Algorithms

It is critical to use modern technology to combat respiratory infections in order to provide more convenient service and avoid disease outbreaks in the future. Big data and AI have demonstrated a high level of capability in disease control and diagnosis [97], [98]. The devices will gain a better understanding of virus structure and disease spread by combining both technologies.

#### 3) Levitate the Economics of the Bio-Techs Market

Oracle cloud computing through its data analysis tool assisted in the development of vaccines against a variety of respiratory diseases which would be beneficial from both an economic and scientific standpoint. Recent findings indicate that 5G wireless technologies e.g., drones, IoT and localization can be used to combat the COVID-19 pandemic.

#### 4) More Secure Platforms

As patient data is extremely vulnerable to leakage, the government must earn the trust of its citizens to share their data with healthcare systems. As a result, it is very important to improve technology’s confidentiality and dependability by implementing secure solutions, policies and bans.

## VI. Conclusion

This paper attempted to provide a comprehensive review of the valuable contributions made by a wide range of professionals working on the frontlines such as hospitals, clinics and laboratories, as well as researchers and scientists. AI implementation has made a significant contribution to the digital health field by utilizing the fundamentals of Automatic Speech Recognition (ASR) and deep learning algorithms. We discussed several methods for distinguishing cough sounds from other cough sounds in this paper including DNN, RNN, ANN, and k-NN. We created a taxonomy to distinguish between papers that included more datasets in testing their algorithm. From the analysis we discovered that the most used technique in respiratory diseases detection and diagnosis methods is the SVM classifier along with LR, while ANN is widely used as compared to RF and DNN. Furthermore, we discussed some of the field’s challenges as well as some solutions and recommendations to interested parties for developing and funding the medical technology sector. As future work, we intend to study the utilization of big data analytics tools along with AI approaches to diagnose more severe infections and control their spread in a timely manner.
